# The Impact of Endocrine Disruptors on Endocrine Targets

**DOI:** 10.1155/2013/340453

**Published:** 2013-06-27

**Authors:** Ewa L. Gregoraszczuk, Radmila Kovacevic

**Affiliations:** ^1^Department of Physiology and Toxicology of Reproduction, Institute of Zoology, Jagiellonian University, 31-007 Krakow, Poland; ^2^Department of Biology and Ecology, University of Novi Sad, Faculty of Sciences, Novi Sad, Serbia

The potential effect of the so-called endocrine disruptors (EDCs) or xenoestrogens on human health and the proven effect on wildlife have got considerable attention in the scientific community. Endocrine disruption represents one of the most controversial environmental issues despite the fact that many substances, both natural and artificial, have been recognized to interfere with endocrine signaling pathways. Such interactions have been documented both in laboratory animal studies as well as *in vitro*. However, in humans there is limited evidence of endocrine disruption caused by EDCs. EDCs are a large group of persistent organic pollutants (POPs), such as polychlorinated dibenzo-p-dioxins (PCDDs) and dibenzofurans (PCDFs), polychlorinated biphenyls (PCBs), and polybrominated ethers (PBDEs), chloronaphtalenes (PCNs), and bisphenol A (BPA), stable, lipophilic pollutants that affect fertility and cause serious reproductive problems. Xenoestrogens are synthetic compounds, but there are also numerous natural molecules in food that exhibit estrogen-mimetic activities. These natural molecules are mainly phytoestrogens isoflavones, and the most consumed are genistein and daidzein. Additionally, certain mushrooms or fungi can contain estrogen-like compounds called mycoestrogens.

Xenoestrogens activities in oocyte, ovary, placenta, and mammary gland and in the consequent serious reproductive problems, including ototoxic action, lack of ovulation, premature ovarian failure (POF), or polycystic ovarian syndrome (PCOS) are discussed in the review E. Gregoraszczuk and A. Ptak “*Endocrine-disrupting chemicals: some actions of POPs on female reproduction.*”

G. Kerdivel et al., in their paper “*Assessment and molecular actions of endocrine-disrupting chemicals that interfere with estrogen receptor pathways*” discuss different molecular actions of some of the major xenoestrogens found in food or the environment and summarize the current models used to evaluate environmental estrogens. This paper is accompanied by clinical study of Caserta et al. “*Correlation of endocrine disrupting chemicals serum levels and white blood cells gene expression of nuclear receptors in a population of infertile women*” compares the internal exposure to bisphenol A (BPA), perfluorooctane sulphonate (PFOS), perfluorooctanoic acid (PFOA), monoethylhexyl phthalate (MEHP), and di(2-ethylhexyl) phthalate (DEHP) in serum samples of 111 infertile women and 44 fertile women and analyses levels of gene expression of nuclear receptors (ER*α*, ER*β*, AR, AhR, PXR, and PPAR*γ*) as biomarkers of effective dose.

Two of the papers deal with aspects of alkylphenols action as endocrine disruptors. 

The paper by B. Yi et al. “*Association between endocrine disrupting phenols in colostrums and maternal and infant health*” showed that most neonates who are exposed to BPA rather than NP or OP via colostrum are recommended continuous biomonitoring of the phenols to clarify their suspected health risk on neonates and pregnant or gestation mothers. Furthermore, A. Hejmej in their paper “*Photoperiod-dependent effects of 4-tert-octylphenol on adherens and gap junction proteins in bank vole seminiferous tubules*” evaluating *in vivo* and *in vitro* effects of 4-tert-octylphenol (OP) on the expression and distribution of adherens and gap junction proteins, N-cadherin, *β*-catenin, and connexin 43 (Cx43), in testes showed that long-term treatment with OP resulted in the reduction of junction proteins expressions independent of FSH indicating that OP acts directly on adherens and gap junction proteins in the testes.

I. Wocławek-Potocka et al. in their paper “*Diverse effects of phytoestrogens on the reproductive performance: cow as a model*” review how exposure of soybean-derived phytoestrogens can have adverse effects on reproductive performance in female adults. Authors suggest that these findings should be specially taken into consideration when recommendations are made regarding dietary or therapeutic phytoestrogen intake in humans. Particularly that they are commonly recognized as therapeutic compounds.

Polychlorinated naphthalenes (PCNs) are new player as endocrine disruptors. Data concerning their potency and action on ovarian function are scarce. Dr J. Barc et al. in their paper “*Action of halowax 1051 on enzymes of phase I (CYP1A1) and phase II (SULT1A and COMT) metabolism in the pig ovary*” describe local ovarian metabolism of PCNs in ovarian tissue and suggest that fast activation of phase I enzymes with simultaneous inhibition of phase II enzymes indicates that androgenic action of PCNs on follicular steroidogenesis may partially result from metabolite action occurring locally in ovarian follicles.

To complete the issue, B. Ayala-García et al. revise current knowledge about the epigenetic mechanisms that underlie the effects of EDs on phenotypic variability and plasticity to stress the value of using the information derived from experiments with EDs to unveil the mechanisms that underlie phenotypic variability and speciation through epigenetic phenotypic plasticity.

Taking into account that in this special issue have been published review articles, research articles and clinical studies, we hope that the information published in this special issue enriches the knowledge of our readers and scholars interested in the influence of xenobiotics on human health.

*Ewa L. Gregoraszczuk* *Ewa L. Gregoraszczuk* *Radmila Kovacevic* *Radmila Kovacevic*

